# Using telehealth to support community health workers in Uganda during COVID-19: a mixed-method study

**DOI:** 10.1186/s12913-023-09217-w

**Published:** 2023-03-27

**Authors:** Maarten Olivier Kok, Tosca Terra, Raymond Tweheyo, Marinka van der Hoeven, Maiza Campos Ponce, Marceline Tutu van Furth, Elizeus Rutebemberwa

**Affiliations:** 1 Erasmus School of Health Policy and Management, Erasmus University, Rotterdam, The Netherlands; 2 Healthy Entrepreneurs Foundation, Kampala, Uganda; 3 Department of Health Policy Planning and Management, Makerere University, Kampala, Uganda; 4 Amsterdam Public Health Research Institute, Amsterdam, the Netherlands; 5 Amsterdam UMC, Vrije Universiteit Amsterdam Infectious Diseases, Amsterdam, The Netherlands

**Keywords:** Telehealth, COVID-19, Community Health Workers, Call-center, Micro Entrepreneurship, Social Entrepreneurship, Uganda

## Abstract

**Background:**

At the onset of the COVID-19 pandemic, a local consortium in Uganda set up a telehealth approach that aimed to educate 3,500 Community Health Workers (CHW) in rural areas about COVID-19, help them identify, refer and care for potential COVID-19 cases, and support them in continuing their regular community health work. The aim of this study was to assess the functioning of the telehealth approach that was set up to support CHWs during the COVID-19 pandemic.

**Methods:**

For this mixed-method study, we combined analysis of routine consultation data from the call-center, 24 interviews with key-informants and two surveys of 150 CHWs. Data were analyzed using constant comparative method of analysis.

**Results:**

Between March 2020 and June 2021, a total of 35,553 consultations took place via the call center. While the CHWs made extensive use of the call center, they rarely asked for support for potential Covid-19 cases. According to the CHWs, there were no signs that people in their communities were suffering from severe health problems due to COVID-19. People compared the lack of visible symptoms to diseases such as Ebola and were skeptical about the danger of COVID-19. At the same time, people in rural areas were afraid to report relevant symptoms and get tested for fear of being quarantined and stigmatized. The telehealth approach did prove useful for other purposes, such as supporting CHWs with their regular tasks and coordinating the supply of essential products. The health professionals at the call center supported CHWs in diagnosing, referring and treating patients and adhering to infection prevention and control practices. The CHWs felt more informed and less isolated, saying the support from the call center helped them to provide better care and improved the supply of medicine and other essential health products.

**Conclusions:**

The telehealth approach, launched at the start of the COVID-19 pandemic, provided useful support to thousands of CHWs in rural communities in Uganda. The telehealth approach could be quickly set up and scaled up and offers a low cost strategy for providing useful and flexible support to CHWs in rural communities.

## Background

On March 11^th^, 2020, the World Health Organization declared the COVID-19 outbreak a global pandemic. At that time, there were 134,576 reported cases and 4981 deaths worldwide [1]. On March 22^nd^, 2020, Uganda confirmed its first COVID-19 case [2]. Soon after, the Ugandan Government instituted a range of nationwide measures that included closure of all non-essential businesses, public transport and schools [3, 4]. While mitigation strategies were expected to slow transmission, modelers predicted that the COVID-19 pandemic would rapidly overwhelm the health system [5].

When it became clear that the pandemic would reach the African continent, many in the Ugandan health sector were deeply concerned [6, 7]. While the Ugandan health system had experience in dealing with outbreaks of infectious diseases, such as Ebola and Cholera, the public health sector was chronically underfunded and understaffed, especially in rural areas [7]. The doctor-patient ratio was only 4% of what is recommended by the WHO and most health professionals worked in a few large cities. There was little protective equipment in stock, and there were only 55 ICU beds available, of which 83% were located in Kampala city and 75% in private hospitals, for a country of approximately 44 million people [8].

A key lesson from recent Cholera and Ebola outbreaks in the region was that it was difficult to simultaneously manage an outbreak and maintain essential health services [9]. During previous outbreaks, a lot of staff was needed to quickly inform the population and identify, isolate and treat possible cases [10–12]. Regular care and outreach activities had to be scaled down and patients stayed away because they feared being infected or couldn't reach health facilities. Studies showed that the reduced access to essential care could have dramatic consequences, ultimately leading to more morbidity and mortality than the virus outbreak itself [10, 13]. The problems with reduced access to health services were most severe in rural areas, where there was already a severe shortage of doctors and nurses, and Community Health Workers (CHW)s played a significant role in providing basic health services [14, 15].

In preparation for the COVID-19 pandemic, health workers across the country had to learn how to identify and care for potential COVID-19 cases, prevent the virus from spreading in the community and protect themselves [16]. An impending challenge was to quickly inform thousands of CHWs in rural and remote areas about the new virus, while the country went into lockdown and public life and transport were shut down. In addition to informing them, a way had to be found to support the CHWs during the pandemic and continue to supply them with essential health products [12, 17]. The role of CHWs was considered important as studies have shown that, during an outbreak, well-informed and locally embedded CHWs can play an important role in educating and mobilizing local communities, identifying and referring potential cases, preserving trust and ensuring access to essential primary health care [9, 12, 16].

In close collaboration with the Ministry of Health, a local consortium developed a telehealth approach that aimed to inform CHWs about COVID-19, support them in identifying, referring and managing cases and help them to continue to provide basic health services and products in their communities. In Kampala, a call-center was set up with a team of nurses and doctors who supported a network of approximately 3,500 community health workers operating in 23 rural districts. These CHWs were part of the regular public health system and were simultaneously supported by a non-profit organization called Healthy Entrepreneurs (HE) [18, 19]. HE uses an approach that draws upon the principles of micro entrepreneurship to support CHWs who are active in rural and remote communities [19]. These CHWs continued their standard community health activities within the public system, while making a modest income from selling basic health products such as malaria medicine, ORS, contraceptives and soap. These CHWs were equipped with a mobile phone and tablet and met in monthly cluster meetings, where they received further training, discussed patients, and were supplied with the products which they had ordered.

The telehealth approach supported the CHWs through a toll-free line, with a team of health professionals who reached out to the CHWs to inform them about COVID-19 and the support that was available. The team offered up-to-date information about COVID-19, triage for identifying suspected cases and advice about isolation and prevention measures and referrals. Besides identifying and monitoring suspected COVID-19 patients, the team could also support consultation on conditions such as malaria, pneumonia, diarrhea, sexual and reproductive health, diabetes and hypertension. In many countries in Sub-Saharan Africa, CHWs play an important role in providing access to basic health services and products for people in rural communities [20–22]. While there are several studies on the use of telehealth, most existing studies focus on direct telehealth support provided to patients in high-income countries [23–26].

In this study, we define telehealth as the use of telecommunication technologies to support and promote long distance clinical care, patient and professional health related education, public health and health administration [22, 23]. The few studies on telehealth conducted in Sub-Saharan Africa mainly focus on contact tracing [27–30], highlighting the potential of telehealth and practical challenges such as the lack of technology and infrastructure, the lack of a funding mechanism, and the possible bias of patients against telehealth [23]. Little is known about the efficacy of using telehealth to support CHWs providing basic health services and products in rural and remote communities [31]. Insight into how telehealth can be leveraged to support CHWs is highly relevant, as it may provide a flexible, relatively inexpensive and scalable approach to rapidly assist thousands of CHWs and help improve access to health care for millions of people.

The aim of this study was to assess the functioning of the telehealth approach that was set up to support Community Health Workers (CHW) in rural communities in Uganda with sustaining basic health services and dealing with the COVID-19 pandemic.

## Methods

### Study design

For this mixed-method study, we combined analyses of routine data about all the calls that were made to the call-center, in-depth interviews with purposively selected key-informants and two surveys of 150 active CHWs.

### The telehealth-approach

The call-center was set up in close collaboration with the surveillance team of the Ministry of Health (MOH) Uganda. The health professionals working at the call-center used the COVID-19 risk profile protocol from the Ugandan MOH to identify low, medium and high risk COVID-19 cases. The protocol identified people with symptoms of COVID-like illness and those with exposure to someone with COVID or travelled to a high risk area. Symptoms were defined as a fever 100.0°F, difficulty in breathing and cough. In case COVID-19 was ruled out, the triage model could be used for identifying other disease symptoms.

**Fig. 1:**
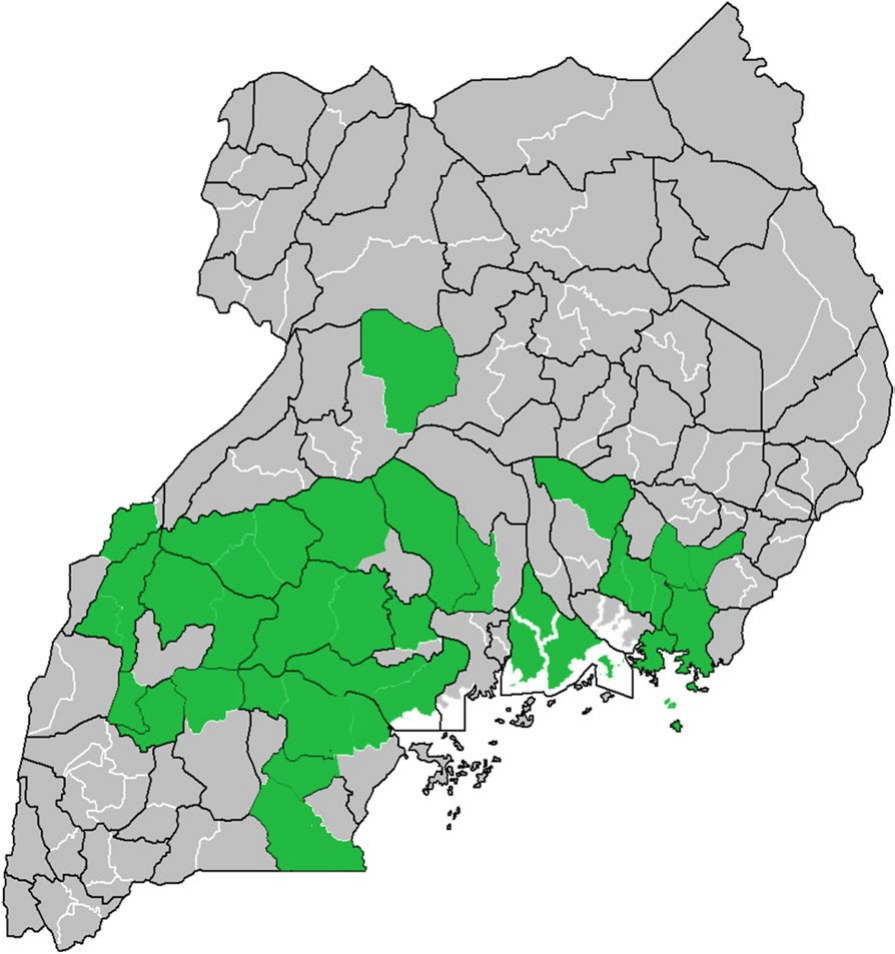
The 23 districts in which the supported CHWs are active

The call-center supported a network of approximately 3500 CHWs in rural communities, whereby the distance to the health facility is at least 7 km. These 3500 CHWs had participated in cluster meetings and been actively supplied during the 6 months before the start of the pandemic. Figure 1 shows the 23 districts in which the CHWs are active. Each CHW reaches an area averaging 700 community members. In total, the CHWs in this network serve an estimated 2.450.000 people. The call-center was set up in Kampala, with 4 local nurses and 2 clinical officers who were trained by an external party on providing teleconsultations.

Upon calling the toll-free line, the CHWs receive a standard message with information on COVID-19. The caller was redirected to one of the health experts to perform a triage. If the caller reported a patient who identified as a medium or high risk COVID-19 case, immediate referral to the respective district surveillance team was made. Clear communication channels between the call-center and the district surveillance teams were established to ensure follow-up and management of potential COVID-19 cases in the districts. The line was activated on March 23 2020 and the call center has since remained open for seven days per week, from 7:00 to 23:00 from Monday to Friday, and from 9:00 to 18:00 on Saturday and Sunday.

### Data collection

#### Incoming calls and the topic of the tele-consultations

Basic data about calls (duration, phone number) to and from the call-center were automatically registered in the XCally system. After approval of the CHW, the health worker at the call-center registered the reason for calling in a separate system. The consultations were categorized in; 1) related to COVID-19, 2) health related, excluding COVID-19, 3) question that were not health related, such as information about cluster meetings, training and logistics, 4) not specified.

### In-depth interviews with key-informants

To gain insight in the functioning of the telehealth-approach and the experiences and perceptions of those involved, we conducted in-depth interviews with purposively selected key-informants, who worked at the call-center (*n* = 4) or as community health worker (*n* = 20). In November 2020, three researchers interviewed 4 health professionals who worked in the call center. One interviewer (TT) conducted the interview face-to-face in Uganda, while two other interviewers (MK and MvH) participated by Skype. These interviews were conducted in English, used a topic list and lasted between 60 – 90 min. The topic list included 5 sections: call-center usage, patients and symptoms, COVID-19 in the community, myths and believes and testing capacity. Next, the research team specified the topic list for semi-structured interviews with CHWs who had used the call center. For these interviews, 20 CHWs were selected, with one from each district, using a list of CHWs who had used the call-center at least once. Interviews were conducted over the phone in local languages by a Ugandan researcher and lasted between 30–45 min. After approval, all interviews were recorded and transcribed verbatim, either in English, or in the local language and translated to English by independent translators.

### Survey among CHWs to explore their experiences with telehealth services

To gain further insight into the perspective of the CHWs on the usefulness of the telehealth approach and better understand why there were so few calls about COVID-19, we twice surveyed the same group of 150 active CHWs, in November 2020 and in March 2021.

The short survey was developed by the research team and consisted of 13 questions; 5 questions on their work as CHW, 5 questions on the usage of the call-center, 3 questions on disease symptoms in the community. The short survey was translated from English into the local languages Luganda, Lusoga and Runyankole and subsequently back-translated to confirm unambiguous and valid construction of the survey in both languages. For these surveys, we selected 150 active CHWs from all districts. We classified CHWs as active if they had continued to request supplies in the six months prior to the interviews, regardless of whether they had ever used the call center. According to these criteria, 84% of CHWs was classified as active. Using the list of active CHWs, the interviewers randomly selected respondents from each district for the survey. CHWs were called over the phone in the local language and answers were filled in by a Ugandan researcher using the online ODK form.

### Data analyses

The translated interview transcripts were coded using NVivo 12 software by the first author (MK) and two research assistants, who coded a subset of the interviews in parallel. Differences in coding were discussed until a shared understanding was reached. Using constant comparative method of analysis [32], two researchers (MK, TT) identified and discussed the emerging themes from the coded interview transcripts. The emerging themes were combined with the results of the survey data to develop a rich and coherent narrative of the functioning and use of the telehealth approach, perspectives on the support provided by the call-center and perspectives on the COVID-19 pandemic in rural communities.

### Ethical clearance

Ethical clearance was obtained from the Makerere University Higher Degrees, Research and Ethics Committee (HDREC), (Ref: Protocol 782) and the Uganda National Council for Science and Technology (Ref: HS955ES). All activities of the CHW-support center were agreed upon by the Director General of the Ministry of Health in Uganda.

## Results

### The CHWs in the network

In Table 1 we describe the basic characteristics of the 3500 CHWs who were connected to the call-center, the 24 purposively selected participants who were interviewed and the 150 CHWs who participated in the first survey in November 2020, of which 123 participated in the second survey in March 2021.

**Table 1:** Summary characteristics of CHWs and study participants

**The CHWs in the telehealth network (*n*** **= 3500)**	
Age (years), mean (SD)	39.15 (8.8)
Female, *n* (%)	2519 (72%)
People reached in area, mean (SD)	696 (715)
**Interview participants (*n* = 24)**	
**Nurses (*n* = 3) and clinical officer (*n* = 1) at call center**	
Age in years, mean (SD)	27,3 (3.2)
Female, n (%)	2 (50)
**Community Health Workers (*n* = 20)**	
Age in years, mean (SD)	38.4 (7.3)
Female, n (%)	16 (80)
**CHWs participating in survey (*n* = 150)**	
Age (years), mean (SD)	42.6 (26.5)
Female, *n* (%)	134 (89.3)
Years active as a CHW, mean	10 (5.2)

### Incoming calls and support from the call-center

The call center started to operate on 23^rd^ of March 2020. In the first two weeks, all CHWs were contacted by the call-center to make them aware of the support that they could get and to provide them with information about COVID-19. CHWs were called directly by the health workers at the call-center and were send text messages in which CHWs were asked to listen to pre-recorded information about COVID-19, which was available in various local languages. To further promote the use of the call center and share information about CO VID-19, flyers and poster were printed and distributed among all CHWs during their monthly cluster meetings.

### The support provided by the tele-health approach

**Fig. 2:**
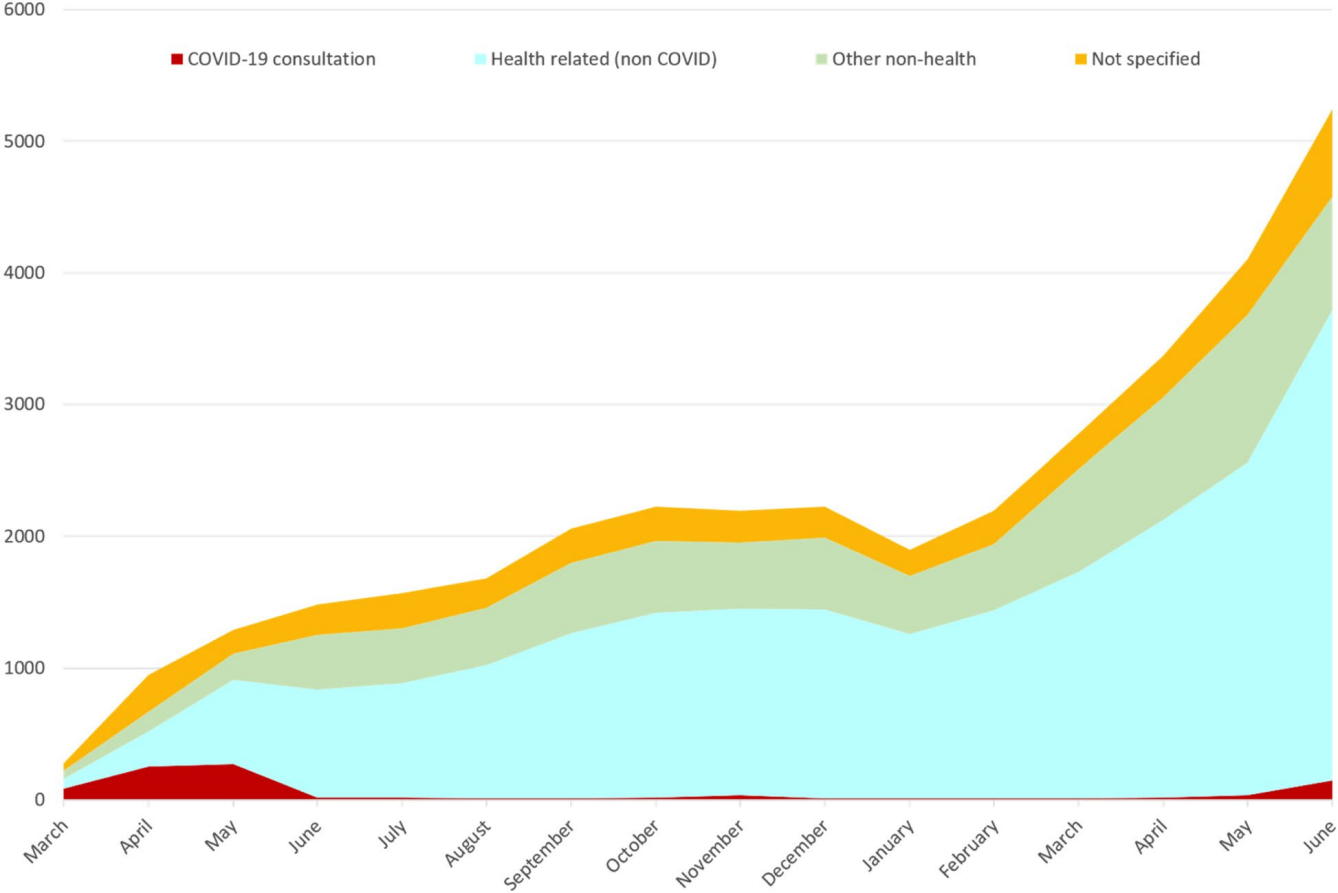
The number and topics of calls from March 2020 until June 2021

Throughout its first year, the number of incoming calls per month steadily increased (Fig. 2). From 23^rd^ March 2020 until the end of June 2021, the CHWs made 35,553 calls to the call-center. In total, 82% of the CHWs had used the call center and 71% used it more than once. CHWs who called multiple times, called on average 14 times to the call-center.

### Use of the call-center by CHW (*N* = 3500)

The call center was intended to inform CHWs about COVID-19, assist CHWs facing potential COVID-19 cases and support them with sustaining regular community health services. While at the start, COVID-19 was expected to be an important issue for the call center, during the first 16 months, only 2.6% of the calls received were about COVID-19 and only 5 potential COVID cases were reported to the call center.

As Fig. 1 shows, the majority of the calls about COVID-19 (61%) came in during the first two months of the pandemic. During these first months, many CHWs had questions about COVID-19 and called to ask whether the disease was real, how it spread, why there were no infections in Uganda and what they could do to stay safe and stop it spreading in their communities. Several CHWs were afraid to continue their work by fear of contracting COVID-19. The nurses and doctors at the call-center tried to counsel these CHWs on infection prevention and control practices and arranged that all CHWs were send a set of personal protective equipment through their monthly cluster meeting.

### Infections and perceptions

To better understand why there were so few calls about COVID-19, while the total number of incoming calls increased each month, we interviewed the staff of the call-center and 20 active CHWs and surveyed 150 active CHWs twice. These 150 CHWs served about 104,400 community members in rural communities in 23 different districts.

### Few signs that COVID-19 is causing severe health problems

Most of the surveyed CHWs (94%) reported that, until March 2021, there had been no signs that COVID-19 was causing severe health problems in their communities. While 9 out of 150 CHWs (6%) had seen patients with relevant symptoms in their communities, these symptoms tended to be mild and it mostly remained unclear whether these were caused by COVID-19. Only 7 out of 150 (5%) CHWs reported that people in their communities had been tested for COVID-19. According to these CHWs, in total, 74 tests had been conducted since the start of the pandemic, which had resulted in 6 confirmed COVID-19 cases. All CHWs said that they would be aware if people in their communities would suffer from severe COVID-19 symptoms, such as severe respiratory distress.

In the interviews, several respondents pointed out that there could be people who had only mild symptoms, who were not identified as potential COVID-19 cases. CHWs and the staff at the call-center explained that it was difficult to identify mild and moderate COVID-19 cases, as the symptoms were very similar to those of common community illnesses.

“In terms of presentation, it presents as the common community illnesses that we have. They may receive a COVID suspect, but they do not have the ability to differentiate between COVID and other common presentations. So, they will treat it as a flu, they will treat it as a cough.” – nurse 1.

### Questioning the existence and danger of COVID-19

Interviewees emphasized that the Ugandan government had tried to educate people about COVID-19, but struggled to reach those living in rural communities. According to the CHWs, a substantial minority of the population questioned whether COVID-19 existed and an even larger group did not consider it to be dangerous. An important reason for questioning the existence and/or danger of COVID-19 was that there were no concrete signs that COVID was causing severe health problems or deaths in their communities.

“The biggest influence on our communities not taking up the COVID threat has been the fact that we had less deaths. To the government and to us, the health workers, it was well managed. To the communities this was actually negative, in such a way that they lost believe that actually this disease does exist. Most of them up to right now believe that actually this disease does not exist. Some of them believe that their bodies are able to fight this infection and so there is no need to get worried like they used to.” – clinical officer 1.

Several participants said that the lack of concrete experience with severe health problems was the key reason for skepticism towards COVID-19.

“Most people do not go to school, they believe in first seeing and experiencing. If they have not experienced it, in most cases, then it’s hard to believe it. If we had lost a number of people, the death cases like the European countries, I am pretty sure that the response of the communities would be quite different.” – CHW 1.

Participants explained that people compared the COVID-19 pandemic with their previous experiences with outbreaks of Ebola and Cholera.

“The biggest percentage of the population believe that actually this virus could not be compared to the previous outbreaks we had the Ebola, the Cholera, which could kill in a few hours. If you look at cholera outbreaks, they happened mostly in the rural areas. People believe that actually, this virus does not kill. You can actually live with it.” – clinical officer 1.

Participants described how the perception of COVID-19 had evolved since the start of the pandemic. In the first few months, people were more concerned about the new virus. Over time, people had become more skeptical about the threat that the virus posed to their communities.

“At first people were so much inquisitive. They did not know about this and so they wanted to know that much. But at the time, when they realized we could co-exist with the infection, maybe it’s not true, probably they have been lied to. So, the inquisitiveness, the eagerness to know much about this disease, to inquire about this disease also reduced. That explains the fall in the number of calls,” – nurse 2.

In addition to the absence of experiences with severe health problems, CHWs also pointed to incidents and stories that had reinforced disbelief in COVID-19.

“I remember at the beginning of the lockdown a few religious leaders also did not believe COVID existed. As you know, in Africa, religion actually kind of affects most people in terms of belief. When a religious leader stands up and says probably this is not real, then you find that he will take along with him a number of people”. – nurse 1.

A common explanation for questioning the existence and/or threat of COVID-19 was that people believed that the COVID-19 threat was an attempt by the ruling powers to manipulate the upcoming elections. Several interviewees mentioned that community members believed that the threat of COVID-19 was made up to avoid people going voting.

“Most of the stories relate to the current elections and that the COVID-19 cases were political, because we are nearing the election. So, they think the ruling party is trying to scare the communities not to go to polling stations and vote” – nurse 2.

A final reason for questioning the COVID-19 threat was that both ideas about how it spreads, and preventive measures such as social distancing were at odds with the local culture that emphasizes togetherness.

“In our communities, most of the populations are illiterate, they are not educated. It’s quite difficult for a village person who was not going to school to accept, about an infection that can spread to humans through sitting near to his relatives, because the culture, the social way of life promotes togetherness.“ – CHW 4.

### Fear for quarantine and stigma

Several interviewees said that people did not want to report COVID-19 related symptoms and did not want to be tested, because they were afraid of being quarantined and stigmatized.

“People fear it so much, that is why many are afraid of testing.” – CHW 4.

The fear for quarantine was confirmed in the second survey in March 2021: 89% of the CHWs reported that people in their communities were afraid to be quarantined.

Interviewees explained that the fear for quarantine had emerged during the beginning of the pandemic, when all suspected cases, including those who were not yet tested, were picked up by health officials and quarantined out of precaution.

“Potential suspects were transported to the isolation facilities, where the staff pick up samples and send them for testing in Kampala. Until the results returned, either positive or negative, the patient was retained. As long as they were suspects they would keep them in the facilities for two weeks”. – clinical officer 1.

CHWs gave multiple reasons why people feared quarantine: people did not want to be taken away from their community to facilities that were far away, they did not know where they would be taken and for how long, they were afraid to have to pay a lot of money and did not want to be locked up with others who could infect them. Several interviewees pointed to news reports and stories on social media that claimed that people were treated badly in the facilities.

“This is seen even on the news. A few of the victims complaining of the low level care that is being given to them, no food, they are not given much attention in terms of welfare, accommodation.” – CHW 2.

A medical doctor working at the call center explained that, while in the large cities the facilities for COVID-19 cases seem to function well, health authorities in rural districts had struggled to set up and manage basic facilities in which potential COVID-19 cases could be quarantined. Rumors about poorly organized facilities were confirmed by people who had been quarantined and had returned to their communities.

“People definitely hate and fear quarantine because those that have ever been tell them that they’re suffering, they tell them that they dump them like prisoners without even providing food” – clinical officer 1.

Besides fear of quarantine, interviewees said people were afraid of being associated with COVID-19 and being stigmatized and excluded as a result.

Since the start of the pandemic, the fear of being associated with COVID-19 had been a recurring issue in the interactions between the CHWs, local communities and the health workers at the call-center. A teleconsultation at the beginning of the pandemic provides an illustrative example. On May 25^th^, 2020, a CHW contacted the call-center and reported the first suspected COVID-19 case. The nurse at the call-center who handled the case:

“there was a CHW that called in from Mityana [district] with a suspected case who travelled from Sudan, and we suspect she might have COVID. The CHW asked: what to do about it? I told them to refer the patient for further checkups. The CHWs was very negative about this advice. She said they will see me as a bad person who is trying to stigmatize their child.” – nurse 1.

When returning back to her community the CHW was blamed by the community members for reporting the case.

“there was a negative perception from the general community towards this CHW who had played a part in identifying this case. They thought it was wrong having reported the case that led to picking of this person and isolating this person”. – nurse 1.

Several CHWs described that the fear of being associated with the disease led people to conceal relevant symptoms.

“I am very sure that there are people who are still concealing their health status and may be a risk to others and they conceal declaring that they are actually not feeling well” – CHW 6.

Community members do not want any contact with infected people or with people that had been in contact with them. Interviewees describe that it does not matter whether someone is declared COVID-19 free, the stigma remains:

“They don’t want to associate with him or anybody that has come into contact with

*them”* –* nurse 2*

Stories of how healthcare facilities in rural areas dealt with COVID-19 cases seemed to amplify the fears of being associated with COVID-19. A medical doctor from the call-center described how staff at a rural facility felt unprepared and tried to turn away a case.

“And so, when they received the case, people obstructed the ambulance from reaching the hospital. Including the member of parliament himself, saying the hospital staff have not been empowered, have not been trained, have not been prepared enough to receive such a case” – clinical officer 1.

### Supporting regular community health work

Besides informing CHWs about COVID-19 and helping them to identify, refer and support potential COVID-19 cases, the other key aim of the telehealth approach was to support the continuation of regular community health work during the pandemic.

The data from the call-center, interviews and surveys indicate that the telehealth approach provided a useful strategy for supporting CHWs in rural communities with the provision of basic health services and products. Since the launch of the call-center, the number of teleconsultations increased each month, from 850 in April 2020 to over 5200 in June 2021.

CHWs who used the call center shared how it supported them: the call center gave them quick and free access to the expertise of a nurse or doctor who could help with triage, diagnostics and patient referrals and answer questions about health issues and products. The CHWs pointed out that they often worked in isolation and it was practically impossible to consult with a healthcare professional. The call center provided a useful helpline.

‘’It helps a lot… It still brings the health services nearer to the people who are not close to health centers or hospital” – CHW 7.

CHWs said that the call center gave them the feeling that they could receive support if needed and that they could provide better care especially when they were in doubt about a patient. The CHWs also said that the direct line to trained health professionals strengthened their position as CHW in the community. In the survey, 77.3% of the CHWs answered that the call center support improved the quality of their work. Of those who had used the call center, 93.2% felt they were getting the help they needed to treat their patient.

### Sustaining the supply of medicine and health products during the lockdown

When their supplies ran low, CHWs could also use the call-center to order medicine and other health products, such as ORS, malaria tests and modern contraceptives, which could be delivered to them in monthly cluster meetings. Requesting supplies through the call center was especially useful in the first four months of the pandemic when the country went into lock down. During this period, the CHWs reported a substantial increase in the need for medicine and health products in their communities and ordered 26% more supplies. In the survey, 75% of the CHWs answered that the call-center improved their access to health products.

### Challenges with the use of the call-center

Interviewees pointed out that, although call center use had increased, the telehealth approach had not yet reached its full potential. After one year, 18% of CHW had not used the call center. Interviewees said that CHWs were used to working independently and the lack of physical interaction made it difficult to promote the call center.

“We did this orientation from a distance. This is at a time when we were in lockdown and so we did not have physical contact with the CHWs, which is quite different when it comes to winning trust and people believing in the service and taking it on.” – clinical officer 1.

A second reason why some CHWs do not use the call center is the general skepticism about telephone services.

“Most of us believe by seeing. Your physical presence with such a population counts a lot. This country is full of dynamics where people have been conned, have lost trust through calls and telecommunication. Most of the CHWs will believe on seeing the person they are going to deal with.” – nurse 2.

## Discussion

### Principal results

This study is the first to investigate the functioning of a telehealth approach that was set up to support CHWs in rural communities in Uganda during the COVID-19 pandemic. We found that telehealth is a useful approach to support CHWs who are active in rural communities. Although the looming pandemic was the reason to set up the call center, in the sixteen months covered by our study, COVID-19 played only a limited role in the teleconsultations. At the start of the pandemic, when Uganda went into lockdown, the call center helped provide the CHWs with information about COVID-19 and infection prevention and control practices and answer urgent questions about the new virus. After these initial months, less than 1% of the teleconsultations was about COVID-19 and only a few possible COVID-19 cases were reported to the call center, which could be referred to the district surveillance teams. Although these first experiences suggest that the telehealth support can be useful, the number of potential cases was too small to gain insight into the added value of the telehealth approach for identifying and caring for COVID-19 cases.

The telehealth approach did prove very suitable for supporting CHWs regular health services in rural communities. The call center provided CHWs with quick and free access to the expertise of health professionals who answered questions and offered advice on diagnosing, referring and treating patients. The CHWs felt better informed, less isolated and indicated that the support helped them to provide better care.

During the lockdown and beyond, the call center could also be used to communicate product needs, enabling CHWs to quickly be supplied with essential products such as malaria tests, antibiotics, ORS and contraceptives. By supporting the CHWs, patients in hard-to-reach areas received better services and spent less time and money accessing basic health products and care during the pandemic.

An interesting finding is that the telehealth approach proved useful for multiple purposes. The same technology, skills, call center and network were used to educate CHWs about a new virus, support them in diagnosing, referring and treating patients and coordinating the organization of cluster meetings and the delivery of medicines and other products. The diversity of purposes for which the telehealth approach can be used can help to make it more sustainable, as it offers several reasons to continue to invest in it. At the same time, fulfilling multiple roles can also make it more difficult to organize a telehealth approach effectively. An clear example is the need for triage of inbound calls when the same call center is used for both medical support and ordering medications and other supplies.

Through the interviews and surveys we tried to find out why CHWs rarely contacted the call center for support in identifying, referring and managing possible COVID-19 cases. The CHWs reported almost unanimously that, during the first year of the pandemic, there were no signs that COVID-19 had led to severe health problems in the rural communities in which they operate. The surveys were conducted amongst active CHWs who can be expected to be aware of severe health problems in the communities in which they work.

Our findings are in line with other studies and government data that show that during the first year of the pandemic, the number of symptomatic COVID-19 cases in Uganda has remained relatively small and mostly concentrated in large urban areas[33]. It is unclear what the lack of reports of severe COVID-19 cases says about the actual spread of COVID-19 in rural Uganda. Studies in countries such as Nigeria and Kenya show that the number of people who have SARS-CoV-2 antibodies, and have thus been infected with COVID-19, is far higher than the official estimates reported by national surveillance systems [34, 35]. Uganda has a relatively young population, with approximately 78% below 40, which increases the likelihood that those who are infected are asymptomatic [35]. In addition, there are no seroprevalence studies in rural communities, making it impossible to compare the perceived number of severe COVID-19 cases with other data sources.

The interviews and survey results offer a worrying picture of the perception of COVID-19 in rural Uganda. The lack of visible COVID-19 cases in rural communities, which may have resulted from successfully fighting the first waves of the pandemic, appears to be contributing to skepticism about the danger of COVID-19. The CHWs point out that people in rural communities have been warned for more than a year about a mysterious virus that causes no visible health problems in their immediate environment. At the same time, lockdowns and other preventive measures have a significant negative impact on daily life, such as not being able to attend education, less contact with friends and family, food insecurity, unemployment and impaired access to care [36, 37].

While many people appear skeptical about the danger of COVID-19, there were also signs that people are hesitant to report COVID-19 related symptoms and get tested because they fear being quarantined and stigmatized. People hide their symptoms so as not to be associated with COVID-19. The combination of skepticism about COVID-19 and fear of reporting relevant symptoms makes it more difficult to effectively halt the spread of new COVID-19 variants, identify patients who need help and provide adequate support and treatment.

During the summer of 2021, there has been a sharp increase in the number of hospitalizations for COVID-19 cases in major cities [37]. In the same period, there were reports that Ugandans opt to seek treatment from home to dodge the high fees charged by hospitals. Although the last spike in cases occurred in December 2021, COVID-19 still requires attention. In addition to education about disease outbreaks and stigma reduction initiatives, it will be critically important to address the various root causes for fear of testing, quarantine and hospitalization and mistrust of biomedicine among rural populations [38]. Addressing stigma and discrimination towards people infected with, or recovering from COVID-19, and towards health workers caring for patients with an infectious disease should be a priority.

### Strengths and weaknesses of the study

In this mixed methods study, conducted during the pandemic, we examined in different ways how the telehealth approach functioned in practice and provided support to CHWs. One of the challenges of studying telehealth is that the relevant processes are located in many different places. By using different methods we were able to triangulate information from different sources. The data on the use of the call center provided a basic quantitative picture, the in-depth interviews provided some insight into the story behind the data and the surveys showed to what extent findings from the interviews, such as the fear of quarantine, were shared by a larger number of people. A limitation of the study is that the data on the subject of the calls to the call center data may not provide a completely accurate picture. Call center agents could only fill in one topic per call, while in some calls more than one topic was discussed. A further limitation is that we asked CHWs how people in their communities view COVID-19, rather than the people themselves. While CHWs are part of the rural communities in which they work, they may have their own biases related to their priorities and interests and their experiences as CHWs.

### Implications and future research

In many countries in Sub-Saharan Africa, CHWs play an important role in providing basic care in rural and remote areas [39]. Our analysis shows that, even during a lockdown, it is possible to quickly establish and scale a telehealth approach that provides useful support to thousands of CHWs. While more research is needed, the results could certainly provide inspiration to those trying to use telehealth in other countries to support CHWs.

Existing studies on telehealth in Sub Saharan Africa and elsewhere emphasize that it is difficult to effectively implement and scale telehealth approaches due to a lack of political support, funding and infrastructure and resistance from health professionals [23, 24, 27, 28, 30, 40–42]. The telehealth approach in this study was designed in specific circumstances, which allowed for rapid implementation and scale-up. In Uganda there was a well-functioning network of active CHWs, equipped with a smart phone, and supported during monthly cluster meetings [14, 19]. At the start of the pandemic, many in the public health sector were very concerned about the predicted wave of critically ill patients and decision makers were ready to support telehealth as a solution for informing and supporting CHWs. The implementing consortium had its own funding and facilities and a central organization with health professionals that worked closely with the public health system at the national and district levels.

Those who consider using telehealth to support CHWs elsewhere can examine to what extent similar circumstances exist and how telehealth can best be set up to contribute to health in the specific local situation [43, 44].

In future research, it would be useful to investigate the implementation of telehealth from a multi-level perspective, with more attention for the experiences of patients, reasons for non-adoption of the telehealth service, the interaction with existing health sector and the various layers in the broader institutional environment [45–47]. Given the flexibility and relatively low cost of telehealth, it may also be interesting to explore how telehealth can be used to support CHWs in diagnosing and treating more complex and chronic health problems and thereby help to further improve access to essential care in rural and remote areas. To increase the likelihood that the results of this type of research will be taken up, it makes sense to ensure a prominent role for local research and engage both key decision-makers and the intended beneficiaries [48, 49].

## Conclusion

Our study shows that the telehealth approach provided useful support to thousands of CHWs in rural communities in Uganda. At the start of the pandemic, the call center helped inform CHWs about infection prevention and identifying and referring COVID-19 cases. While the call center was widely used, very few suspected COVID-19 cases were reported.

The telehealth approach did prove useful for supporting CHWs regular health services in rural communities. The call center supported CHWs with diagnosing, referring and treating patients, adhering to infection prevention and control practices, and communicating product needs. The CHWs felt better informed and less isolated and indicated that the telehealth support provided them with better care and timely access to supplies of essential products. The telehealth approach could be set up and scaled up in a short period and appears to be a low cost strategy for providing useful and flexible support to CHWs in rural communities.

## Data Availability

The data generated and/or analyzed during the current study are available from the corresponding author upon reasonable request. The interviews that underlie in this study discuss at times sensitive, locally specific and controversial issues. During our informed consent procedure, we assured participants of anonymity. We are unable to comply with that commitment if we make the interviews recordings available. We believe there is an unacceptable high risk of disclosure in sharing full transcripts, and do not have the resources to redact the interviews fully. However, researchers are welcome to request specific coding queries by contacting the corresponding author. We will run the queries as requested, redact the results only to the extent necessary to ensure anonymity and pass the results on to fellow researchers.

## Abbreviation

CHW: Community Health Worker

## References

[1] Cucinotta D, Vanelli M (2020). WHO Declares COVID-19 a Pandemic. Acta Bio Medica Atenei Parm.

[2] Olum R, Bongomin F (2020). Uganda’s first 100 COVID-19 cases: Trends and lessons. Int J Infect Dis.

[3] Sarki AM, Ezeh A, Stranges S (2020). Uganda as a Role Model for Pandemic Containment in Africa. Am J Public Health.

[4] Amodan BO, Bulage L, Katana E, Ario AR, Fodjo JNS, Colebunders R (2020). Level and Determinants of Adherence to COVID-19 Preventive Measures in the First Stage of the Outbreak in Uganda. Int J Environ Res Public Health.

[5] Walker PGT, Whittaker C, Watson OJ, Baguelin M, Winskill P, Hamlet A (2020). The impact of COVID-19 and strategies for mitigation and suppression in low- and middle-income countries. Science.

[6] Migisha R, Kwesiga B, Mirembe BB, Amanya G, Kabwama SN, Kadobera D (2020). Early cases of SARS-CoV-2 infection in Uganda: epidemiology and lessons learned from risk-based testing approaches - March-April 2020. Glob Health.

[7] Ajari EE, Ojilong D (2020). Assessment of the preparedness of the Ugandan health care system to tackle more COVID-19 cases. J Glob Health.

[8] Atumanya P, Sendagire C, Wabule A, Mukisa J, Ssemogerere L, Kwizera A (2020). Assessment of the current capacity of intensive care units in Uganda. A descriptive study J Crit Care.

[9] Siekmans K, Sohani S, Boima T, Koffa F, Basil L, Laaziz S (2017). Community-based health care is an essential component of a resilient health system: evidence from Ebola outbreak in Liberia. BMC Public Health.

[10] Plucinski MM, Guilavogui T, Sidikiba S, Diakité N, Diakité S, Dioubaté M (2015). Effect of the Ebola-virus-disease epidemic on malaria case management in Guinea, 2014: a cross-sectional survey of health facilities. Lancet Infect Dis.

[11] Keshvardoost S, Bahaadinbeigy K, Fatehi F (2020). Role of Telehealth in the Management of COVID-19: Lessons Learned from Previous SARS, MERS, and Ebola Outbreaks. Telemed J E-Health Off J Am Telemed Assoc.

[12] Bhaumik S, Moola S, Tyagi J, Nambiar D, Kakoti M (2020). Community health workers for pandemic response: a rapid evidence synthesis. BMJ Glob Health.

[13] Hayden EC (2014). Ebola obstructs malaria control: outbreak is shutting down prevention and treatment programmes in West Africa. Nature.

[14] Musoke D, Ndejjo R, Atusingwize E, Ssemugabo C, Ottosson A, Gibson L (2020). Panacea or pitfall? The introduction of community health extension workers in Uganda. BMJ Glob Health.

[15] Brunie A, Wamala-Mucheri P, Otterness C, Akol A, Chen M, Bufumbo L (2014). Keeping community health workers in Uganda motivated: key challenges, facilitators, and preferred program inputs. Glob Health Sci Pract.

[16] Ballard M, Bancroft E, Nesbit J, Johnson A, Holeman I, Foth J (2020). Prioritising the role of community health workers in the COVID-19 response. BMJ Glob Health.

[17] Chandani Y, Noel M, Pomeroy A, Andersson S, Pahl MK, Williams T (2012). Factors affecting availability of essential medicines among community health workers in Ethiopia, Malawi, and Rwanda: solving the last mile puzzle. Am J Trop Med Hyg.

[18] Källander K, Strachan D, Soremekun S, Hill Z, Lingam R, Tibenderana J (2015). Evaluating the effect of innovative motivation and supervision approaches on community health worker performance and retention in Uganda and Mozambique: study protocol for a randomised controlled trial. Trials.

[19] Borst RAJ, Hoekstra T, Muhangi D, Jonker I, Kok MO (2019). Reaching rural communities through ‘Healthy Entrepreneurs’: a cross-sectional exploration of community health entrepreneurship’s role in sexual and reproductive health. Health Policy Plan.

[20] Hartzler AL, Tuzzio L, Hsu C, Wagner EH (2018). Roles and Functions of Community Health Workers in Primary Care. Ann Fam Med.

[21] Perry HB, Chowdhury M, Were M, LeBan K, Crigler L, Lewin S (2021). Community health workers at the dawn of a new era: 11. CHWs leading the way to “Health for All.”. Health Res Policy Syst.

[22] Kok M, Crigler L, Musoke D, Ballard M, Hodgins S, Perry HB (2021). Community health workers at the dawn of a new era: 10. Programme performance and its assessment. Health Res Policy Syst.

[23] Babalola D, Anayo M, Itoya DA, Department of Medicine, College of Medicine, University of Ibadan, Ibadan, Nigeria. Telehealth during COVID-19: why Sub-Saharan Africa is yet to log-in to virtual healthcare? AIMS Med Sci. 2021;8:46–55.

[24] Monaghesh E, Hajizadeh A (2020). The role of telehealth during COVID-19 outbreak: a systematic review based on current evidence. BMC Public Health.

[25] Fisk M, Livingstone A, Pit SW (2020). Telehealth in the Context of COVID-19: Changing Perspectives in Australia, the United Kingdom, and the United States. J Med Internet Res.

[26] Opoku D, Stephani V, Quentin W (2017). A realist review of mobile phone-based health interventions for non-communicable disease management in sub-Saharan Africa. BMC Med.

[27] Manyati TK, Mutsau M, Exploring the effectiveness of telehealth interventions for diagnosis, contact tracing and care of Corona Virus Disease of,. (COVID19) patients in sub Saharan Africa: a rapid review. Health Technol. 2019;2021:1–8. 10.1007/s12553-020-00485-8 PMC7870280 33585154

[28] Kamulegeya LH, Bwanika JM, Musinguzi D, Bakibinga P. Continuity of health service delivery during the COVID-19 pandemic: the role of digital health technologies in Uganda. Pan Afr Med J. 2020;35. 10.11604/pamj.supp.2020.35.2.23115 PMC7875742 33623568

[29] Mogessie YG, Ntacyabukura B, Mengesha DT, Musa MB, Wangari M-C, Claude N (2021). Digital health and COVID-19: challenges of use and implementation in sub-Saharan Africa. Pan Afr Med J.

[30] O’Donovan J, Hamala R, Nalubwama M, Ameniko M, Govina G, Gray N (2021). Roles for mHealth to support Community Health Workers addressing COVID-19. Glob Health Promot.

[31] Källander K, Tibenderana JK, Akpogheneta OJ, Strachan DL, Hill Z, ten Asbroek AHA (2013). Mobile health (mHealth) approaches and lessons for increased performance and retention of community health workers in low- and middle-income countries: a review. J Med Internet Res.

[32] Pope C, Ziebland S, Mays N (2000). Qualitative research in health care. Analysing qualitative data BMJ.

[33] Salyer SJ, Maeda J, Sembuche S, Kebede Y, Tshangela A, Moussif M (2021). The first and second waves of the COVID-19 pandemic in Africa: a cross-sectional study. Lancet Lond Engl.

[34] Okpala OV, Dim CC, Ugwu CI, Onyemaechi S, Uchebo O, Chukwulobelu U (2021). Population seroprevalence of SARS-CoV-2 antibodies in Anambra State, South-East, Nigeria. Int J Infect Dis IJID Off Publ Int Soc Infect Dis.

[35] Wamai RG, Hirsch JL, Van Damme W, Alnwick D, Bailey RC, Hodgins S (2021). What Could Explain the Lower COVID-19 Burden in Africa despite Considerable Circulation of the SARS-CoV-2 Virus?. Int J Environ Res Public Health.

[36] Hamer DH. Short-term and Potentially Long-term Negative Impacts of COVID-19 in Sub-Saharan Africa: Evidence from the Africa Research, Implementation Science, and Education Network Rapid Monitoring Survey. Am J Trop Med Hyg. 2021;:tpmd210617. 10.4269/ajtmh.21-0617 PMC8437182 34161302

[37] Wasswa H (2021). Covid-19: Uganda’s low inpatient numbers mask high community infection as desperate patients turn to herbs. BMJ.

[38] Ndejjo R, Naggayi G, Tibiita R, Mugahi R, Kibira SPS (2021). Experiences of persons in COVID-19 institutional quarantine in Uganda: a qualitative study. BMC Public Health.

[39] Hodgins S, Kok M, Musoke D, Lewin S, Crigler L, LeBan K (2021). Community health workers at the dawn of a new era: 1. Introduction: tensions confronting large-scale CHW programmes. Health Res Policy Syst.

[40] Adebayo PB, Oluwole OJ, Taiwo FT (2020). COVID-19 and Teleneurology in Sub-Saharan Africa: Leveraging the Current Exigency. Front Public Health.

[41] Nguyen NH, Nguyen AQ, Ha VTB, Duong PX, Nguyen TV (2021). Using Emerging Telehealth Technology as a Future Model in Vietnam During the COVID-19 Pandemic: Practical Experience From Phutho General Hospital. JMIR Form Res.

[42] Hajizadeh A, Monaghesh E (2021). Telehealth services support community during the COVID-19 outbreak in Iran: Activities of Ministry of Health and Medical Education. Inform Med Unlocked.

[43] Burchett H, Umoquit M, Dobrow M (2011). How do we know when research from one setting can be useful in another? A review of external validity, applicability and transferability frameworks. J Health Serv Res Policy.

[44] Kok MO, Bal R, Roelofs CD, Schuit AJ (2017). Improving health promotion through central rating of interventions: the need for Responsive Guidance. Health Res Policy Syst.

[45] James HM, Papoutsi C, Wherton J, Greenhalgh T, Shaw SE (2021). Spread, Scale-up, and Sustainability of Video Consulting in Health Care: Systematic Review and Synthesis Guided by the NASSS Framework. J Med Internet Res.

[46] Greenhalgh T, Wherton J, Papoutsi C, Lynch J, Hughes G, A’Court C (2017). Beyond Adoption: A New Framework for Theorizing and Evaluating Nonadoption, Abandonment, and Challenges to the Scale-Up, Spread, and Sustainability of Health and Care Technologies. J Med Internet Res.

[47] van de Bovenkamp HM, Stoopendaal A, Bal R (2017). Working with layers: The governance and regulation of healthcare quality in an institutionally layered system. Public Policy Adm.

[48] Hegger I, Kok MO, Janssen SWJ, Schuit AJ, van Oers HAM (2016). Contributions of knowledge products to health policy: a case study on the Public Health Status and Forecasts Report 2010. Eur J Public Health.

[49] Kok M, de Souza DK (2010). Young Voices demand health research goals. The Lancet.

